# The lived experience of French parents concerning the diagnosis of their children with borderline personality disorder

**DOI:** 10.1186/s40479-024-00258-z

**Published:** 2024-07-01

**Authors:** Léa Villet, Abtine Madjlessi, Anne Revah-Levy, Mario Speranza, Nadia Younes, Jordan Sibéoni

**Affiliations:** 1 Service de psychopathologie de l’enfant et de l’adolescent, Hôpitaux de Saint Maurice, 63 rue de la Roquette, Paris, 75011 France; 2 Service de psychiatrie adulte, Hôpital François Quesnay, Mantes-la-Jolie, 78200 France; 3 Service Universitaire de Psychiatrie de l’Adolescent, Argenteuil Hospital Centre, Argenteuil, 95100 France; 4 ECSTRRA Team, UMR-1153, Université Paris Cité, Inserm, Paris, 75010 France; 5 Service Universitaire de Psychiatrie de l’Enfant et de l’Adolescent, Centre Hospitalier de Versailles, 177 Rue de Versailles, Le Chesnay‑Rocquencourt, 78150 France; 6 Centre de Recherche en Épidémiologie et Santé des Populations (CESP), INSERM UMR 1018 «Developmental Psychiatry and Trajectories», Université Paris-Saclay, Université Versailles Saint Quentin en Yvelines, 16 Av. Paul Vaillant Couturier, Villejuif, 94800 France; 7 Service Universitaire de Psychiatrie pour adultes et addictologie, Centre Hospitalier de Versailles, 177 rue de Versailles, Le Chesnay-Rocquencourt, 78150 France; 8 Université de Versailles, Saint -Quentin en Yvelines, Versailles, France

**Keywords:** Borderline personality disorder, Parents, Diagnosis, Family connections, Qualitative study

## Abstract

**Background:**

Psychiatrists often hesitate to diagnose borderline personality disorder (BPD). While individuals with BPD have reported both positive and negative experiences upon receiving their diagnosis, no study has specifically explored this issue among parents. Parents of children diagnosed with BPD can benefit from recently developed family-support interventions such as the Family Connections program. Our study aimed to explore the experiences of parents learning about their child’s BPD diagnosis and to investigate the impact of the Family Connections program on their experiences.

**Methods:**

This qualitative study, conducted in France following the five-stage IPSE method, involved parents of children with BPD recruited through the Family Connections association in Versailles. We conducted semi-structured interviews and used purposive sampling for data collection until data saturation was reached. Data analysis was performed using a descriptive and structuring approach with NVivo 12 software to elucidate the structure of lived experiences.

**Results:**

The study included 21 parents. The structure of the lived experiences was characterized by three central axes: (1) the long and difficult road to diagnosis; (2) communicating the BPD diagnosis to parents: a necessary step; (3) the pitfalls of receiving the diagnosis. The Family Connections program provided significant support in these areas, particularly in understanding the diagnosis, enhancing communication with their child, and reducing social isolation.

**Conclusion:**

These findings highlight the challenges parents face when receiving a BPD diagnosis for their child and underscore the need for an early, clear, and detailed explanation of the diagnosis. The specific experiences of receiving the diagnosis are indicative of the broader care experience parents undergo and highlight their need and right to be informed, supported, and guided throughout their child’s treatment.

**Supplementary Information:**

The online version contains supplementary material available at 10.1186/s40479-024-00258-z.

## Background

Borderline Personality Disorder (BPD) is a prevalent and complex mental health condition characterized by pervasive instability in emotional regulation, interpersonal relationships, self-image, and impulse control. Treatment for BPD has shown to be effective [1–3]. Informing patients about their illness is not only an ethical responsibility for healthcare professionals but also a crucial step in the management of the condition [4]. However, diagnosing psychiatric conditions is notably different and more complex than diagnosing somatic illnesses for several reasons. Psychiatric conditions are typically considered disorders rather than diseases, as there is no single causal agent that explains a psychiatric syndrome in its entirety [5]. Moreover, psychiatric diagnoses are based on clinical signs grouped into syndromes, which are identified through patient interviews where the clinician’s subjectivity interacts with that of the patient [6]. The approach to psychiatric diagnosis, which involves subjectively defining symptoms as variants of the social norm at a particular time and place, has been long debated by psychiatrists and the anti-psychiatry movement [7].

Studies have revealed that individuals with BPD often receive insufficient information about their diagnosis, leading to experiences of stigma and misunderstanding [8–12]. Despite these challenges, advancements have been made in the diagnostic process, and clinical practice guidelines are now in place to guide professionals [3]. These guidelines recommend providing patients and their families with detailed information about the disorder, including its characteristics, etiological hypotheses, prognosis, and therapeutic options [13–15]. Such transparency helps patients understand their condition and fosters acceptance of their difficulties. Awareness of prognosis and available treatments encourages a therapeutic alliance and active involvement in care.

In recent decades, research, notably the McLean Study of Adult Development, has demonstrated a generally favorable prognosis for most individuals diagnosed with BPD [16]. In France, recent guidelines have been issued on best practices for disclosing psychiatric diagnoses [17], though these are general and not specific to BPD. They are largely informed by studies on schizophrenia [18, 19]. Many mental health clinicians hesitate to diagnose BPD due to concerns about stigma, the potential for increased distress, risks of self-harm, damaging the therapeutic alliance, and a generally pessimistic view of the prognosis [10, 20, 21].

The impact of diagnostic disclosure has been widely studied, highlighting both beneficial and adverse effects [21–24]. Benefits for patients include relief at having a name for their condition, improved understanding of their behavior, enhanced access to resources, psychoeducation, specialized interventions, and stronger therapeutic relationships. Negative impacts can include feeling judged or attacked and experiencing stigmatization and exclusion from healthcare services.

Caregivers, especially parents of individuals with BPD, are profoundly impacted. They frequently report high levels of stress, anxiety, guilt, and feelings of helplessness, which often lead to mental health issues [25]. They also face intra-familial conflicts, social withdrawal, and stigmatization resulting from environmental and financial strains [26, 27]. Their involvement in care should be encouraged with consent from the patient [27–29].

Programs like Family Connections, a peer-led psychoeducational program based on dialectical behavior therapy techniques, have been developed for caregivers of people with BPD [30]. This program aims to increase knowledge about BPD, teach communication skills to support a positive family environment, encourage self-compassion among caregivers, and provide a non-stigmatizing space for family discussions [31]. Evaluations of Family Connections have shown it effectively reduces caregiver burden and improves knowledge, coping strategies, and family dynamics [30, 32–35].

Given the significance of how a BPD diagnosis is communicated, qualitative methods are ideal for exploring this through the experiences of parents. Our study aims to investigate parents’ lived experiences following the disclosure of a BPD diagnosis and assess the impact of the Family Connections program.

## Methods

This exploratory qualitative study used the “inductive process to analyze the structure of lived experience” (IPSE) approach [36]. The IPSE method is based on a descriptive phenomenological approach and relies on an inductive process that probes the lived experience of patients and healthcare professionals in depth and analyzes the structure of their experiences. The research process is divided into five phases, described below.

The research complied with French regulations governing observational research involving the parents of patients (declaration of compliance with the CNIL reference methodology MR004 and entry in the register of such research hosted by the Health Data Hub website). All participants provided informed written consent before inclusion. The report of this study adheres to the COREQ guidelines (see supplementary material) [37].

### Stage 1: Setting up a research group

Our research group included three psychiatrists, each with distinct experience concerning the diagnosis of BPD and its communication. The group also included two psychiatric trainees and a psychologist, all trained in qualitative methods. For heuristic purposes — that is, to enable the discovery of new unknown elements and produce original findings — the group’s members were highly diverse, especially in terms of their knowledge, age, and background. The group continuously practiced reflexivity during open discussions among themselves. Reflexivity can be defined as the researchers’ reflection of their role in the study and its effects on their findings at every step of the research process [38]. In qualitative research, a recurrent hazard is that the findings are close to the reflection or confirmation of the researcher’s preconceptions and beliefs. The process of reflexivity enables researchers to avoid the pitfalls of applying their own assumptions to the material. Throughout the process, all the researchers endeavored to clarify their positions. In practice, they answered these two questions regarding the study: (i) What are my preconceptions and beliefs about borderline personality disorder diagnosis? (ii) What are my expectations regarding this study? This reflexive position was continuously worked on within the group, during open discussions among the researchers. For instance, some authors working in the field of child psychiatry were hesitant to make a formal diagnosis of BPD during adolescence since personality is still in the process of development. Other authors working in adult psychiatry were more accustomed to making this diagnosis with less hesitation. These elements have led to open discussions on the subject.

### Stage 2: Ensuring the originality of the study

Two members of the group systematically reviewed the qualitative and quantitative literature to confirm the study’s relevance and originality. They verified that no qualitative study of this specific topic explicitly focusing on the perspectives of the parents had been conducted. To ensure that the other group members remained inductive and open to novelty, they had access to this review only after they had completed the data analysis.

### Stage 3: Recruitment and sampling aiming for exemplarity

The research group defined the inclusion and exclusion criteria (Table 1) intended to attain exemplarity, that is, to select participants who “have experienced quintessential, typical, or archetypal examples of the situation being studied” [36] and to include participants who might enrich and add new information to previous findings. We thus used a purposive sampling strategy with maximum variation [39], that is, to select parents who differed by sex, age, socio-professional category, and date of participation in the Family Connections program and their children by age, sex, socio-professional category, and type of care. Researchers identified potential participants who they considered likely to provide the most information. In practice, participants were recruited through the mailing list of Family Connections.

**Table 1:** Inclusion and exclusion criteria

Inclusion criteria	Parents
	To have a child diagnosed with a BPD
	To have participated in the Family Connection program
Exclusion criteria	Other family members
	Absence of a diagnosis of BPD for their child
	Refusal to participate in the study

The sample size was not defined in advance but was determined by data saturation according to the principles of “information power”, here, based on the criteria described by the authors: “the quality of the dialogue” during the interview, “the aim of the study”, and the “sample specificity”, that is, “the specificity of experiences, knowledge, or properties among the participants included in the sample” [40]. Inclusion of new participants continued until the analysis of new material no longer yielded new findings. In other words, data collection and analysis were complete when the research group considered that the axes of experience obtained provided a sufficient explanatory framework for the data collected [41].

### Stage 4: Data collection - access to experience

One-to-one interviews were conducted by two psychiatry trainees (one male and one female) who were trained in qualitative methods. Prior to the study, the interviewers had no contact with the participants. They carried out semi-structured interviews using an open-ended approach [42], structured around areas of exploration (Table 2) that were collectively determined by the group after listening to and reading two pilot interviews. The interviews, lasting between 60 and 90 min, were conducted face-to-face or via videoconference, depending on the parents’ preferences. All interviews were audio-recorded and transcribed into anonymized documents that captured the participants’ expressive nuances.

**Table 2:** Interview guide. (translated from French to English for the sole purpose of this article)

Area of exploration	Potential question
Before the experience	What were the first difficulties encountered with your child?
	Could you tell me about your first contacts with the healthcare services?
	How did you experience your child’s difficulties before the diagnosis was communicated?
	What were your expectations in terms of explanations about the diagnosis?
During the experience	In what circumstances did you hear about the diagnosis for the first time?
	Can you describe the communication of the diagnosis?
	How did you feel during the communication of the diagnosis?
After the experience	What was the impact of the diagnosis on you, your child, and their care?
	Why do you think a diagnosis is useful?
	What was the impact of the Family Connections program?
	How do you believe communication of the diagnosis could be improved?

### Stage 5: Data analysis - from the description of the structure of experience to practical implications

The analytical procedure adopted the Interpretative Phenomenological Analysis (IPA) approach. This rigorous method relies on an inductive phenomenological technique, as referenced in source [36]. The analysis comprised two main stages: an individual stage where two researchers worked independently, supported by NVivo software, and a collective stage involving group data analysis.

In the individual stage, the two qualitative researchers independently conducted a systematic descriptive analysis to accurately represent each participant’s experience. This process involved: (1) Listening to each recorded interview twice and reading the transcript three times;

(2) Conducting a word-by-word exploration of the experience, breaking down the entire text into descriptive units; (3)Categorizing these descriptive units into thematic groups. These stages were facilitated by the use of QSR NVivo 12 software.

During the group stage, the researchers regularly convened with other group members who had familiarized themselves with the data by listening to and reading all interviews as needed. These two-hour meetings, which commenced after five interviews had been analyzed, were structured in two sets. The first set focused on the structuring phase, where categories were organized into axes of experience. Each axis was linked to its underlying categories to outline the structure of the lived experiences around central themes. The second set of meetings addressed the practical phase, which included triangulating the data with existing literature to highlight unique findings and suggest practical implications for enhancing care.

Several criteria were employed to ensure the analysis’s rigor, including triangulation, attention to negative cases, and reflexivity.

## Results

The study included 21 parents (6 men and 15 women). All parents who were approached agreed to participate. The characteristics of the participants and children are summarized in Table 3 and those for each participant can be found in the supplementary material (Table S1). The age of the parents ranged from 46 to 69 years and averaged 57.4 years. Participation in the Family Connections program occurred from 2017 to 2022. At the time of the diagnosis, their children with BPD were between 14 and 25 years of age, with an average of 21.4 years. Thirteen of the sons and daughters no longer lived with their parents. Seventeen had been hospitalized several times and 16 took medication. Ten were confronted by the discontinuation of school and unstable employment. At the time of the interviews, one son and five daughters were receiving no or very irregular care.

**Table 3:** Participant characteristics

	Parents	Child with BPD
	*N* = 21	*N* = 20
	*n* (%)	*n* (%)
Sex		
Men	6 (29%)	1 (5%)
Women	15 (71%)	19 (95%)
Age	57.4	26.4
Mean age (years)	(46–69)	(15–34)
Age limits (years)		
Relative mean age at the time of the communication of the diagnosis	Not specified	21.4
Age range		
		(14–25)
Year of participation in the Family Connections program		
2017	3 (14.3%)	
2018	1 (4.8%)	
2019	4 (19%)	
2020	10 (47.6%)	
2021	2 (9.5%)	
2022	1 (4.8%)	
Parental socio-economic category		
High	21 (100%)	
Socio-professional activity of the child with BPD		
Unemployed		10 (50%)
Student		4 (20%)
Out of school		1 (5%)
Non-qualified employment		5 (25%)
Live at parent’s home		
Yes		7 (35%)
No		13 (65%)
Care and treatment		
Psychiatric		13 (65%)
Psychotherapy		7 (35%)
None		6 (30%)
Medication		16 (80%)
Hospitalizations		
None		2 (10%)
1 to 5		11 (55%)
6 to 9		4 (20%)
≥ 10		3 (15%)
Protective measures		
None		15 (75%)
Simple curatorship		1 (5%)
Reinforced curatorship		4 (20%)
National disability allowance		
Yes		9 (45%)
No		11 (55%)

The data analysis revealed a structure of lived experience centered around three main axes: (1) the long and difficult path to diagnosis, (2) the reception of the BPD diagnosis as a necessary step, and (3) the pitfalls of receiving the diagnosis. The transcript excerpts provided below were chosen to illustrate the themes discussed and have been translated into English by a professional scientific translator exclusively for this article. The original excerpts in French can be accessed in the supplementary material (Table S2).

### The long and difficult road to diagnosis

#### The delay in getting a diagnosis

All the parents described a significant delay, often lengthy, between the appearance of the first symptoms and the diagnosis of Borderline Personality Disorder (BPD). During this period, many parents reported that their children had received several other diagnoses, including depression, bipolar disorder, eating disorders, and substance abuse disorder. They described receiving inconsistent information from psychiatrists, with frequent uncertainty between diagnoses.

*P2: “They spoke vaguely about a borderline disorder, then they went back to bipolarity.”*

Some parents reported that no diagnostic hypothesis was shared with them before receiving the BPD diagnosis.

*P4: “Over four years, I can’t remember a single time a diagnosis was even considered as a hypothesis.”*

Others expressed frustration with psychiatrists who refused to provide a diagnosis.

*P1: “The psychiatrist said she was giving medication to test things and she had nothing more to say.”*

Many parents viewed this delay in diagnosis as a sign of incompetence within the healthcare services.

*P2: “We felt they had never read the DSM-5…or the description of BPD…they were able to diagnose bipolarity but not other disorders.”*

From the parents’ perspective, not having an accurate diagnosis meant not receiving appropriate treatment.

P1: “If she doesn’t have a diagnosis, why should she leave with an antidepressant prescription?”

Conversely, some parents recognized the complexities psychiatrists face in making a certain diagnosis, especially during childhood or adolescence. Only one mother explicitly stated that she did not want to receive a diagnosis because she was not ready to accept that her daughter had a chronic illness.

#### Informal diagnosis with little information

Parents reported various ways in which the diagnosis of Borderline Personality Disorder (BPD) was communicated to them. Only a few described a planned or structured approach that provided adequate information and allowed room for questions. Several participants mentioned they did not remember the moment of receiving the diagnosis in detail.

*P4: “He gave us some elements but without many details, at least I don’t remember.”*

Many parents learned about the diagnosis outside of formal consultations. One parent reported that a psychiatrist disclosed the diagnosis after a challenging family therapy session. For another family, the diagnosis was communicated in the corridors of a healthcare facility.

*P5: “It finally happened between two doors—the doctor told me: your daughter has borderline personality disorder.”*

Some parents discovered the diagnosis by chance when it appeared on medical documents needed for administrative purposes.

Before the communication of the diagnosis, several parents had never heard the term “borderline personality disorder,” while others were familiar with the colloquial term “borderline” but did not realize it was the official name of a disorder.

*P12: “I asked the psychiatrist what it meant because I had never heard of it.”*

Parents expressed a desire for a dedicated consultation where they could receive detailed explanations about the characteristics of the disorder, its causes, prognosis, therapy options, and specific support for themselves. Some cited medical overload as a possible reason for the lack of information.

*P6: “I understand the doctor is very busy. He has a full waiting room all day. I understand that he does not have time to explain more.”*

### Communicating the BPD diagnosis to parents: a necessary step

#### The immediate aftermath

Parents highlighted their experiences in the immediate aftermath of receiving the diagnosis. They reported positive reactions: gratitude toward the professional who made the diagnosis and a feeling of relief at being able to name the disorder, as well as a sense of hope from new possibilities to learn more about the disorder and understand what their son or daughter was experiencing.

*P12: “I was grateful to this professional for telling me… this person saved us in a way by giving words to a disorder, because finally someone told me what my daughter had.”*

*P4: “At the time, we didn’t have many explanations, but we got leads to find documents and better understand the disorder.”*

Many parents also felt anxious about the severity of the disorder and worried about their loved one’s future and care, understanding that the disorder was chronic and that medication alone was not sufficient to treat it.

*P8: “There are not many therapists, and there is no medication… Borderline is not the easiest path.”*

It was only after participating in the Family Connections program that the parents gained a better understanding of the BPD diagnosis and, as a direct consequence, noticed an improvement in communication and a calmer relationship with their son or daughter.

*P16: “I think I’m a little more careful in the way I approach her, which is what Family Connections allowed me to do.”*

Parents also reported feeling less powerless after the program. They felt more capable of managing their son’s or daughter’s difficulties, especially in times of crisis, and had the impression of regaining control over the situation.

*P8: “This method allows us to better live with and understand the illness of our loved ones and therefore to adjust our personal attitudes to meet the needs of our loved ones as they evolve.”*

#### A meaningful and helpful diagnosis

Parents reported a gradual acceptance and understanding of the diagnosis after it was communicated.

P5: “I hadn’t fully realized she was sick. It took me a while.”

Some parents discussed the difficulty of grieving the loss of a “normal” child. After receiving the diagnosis, most sought additional information to better understand what it entailed, using various media such as books and videos. Many stated that the Family Connections program was instrumental in helping them understand the diagnosis. Every parent recognized their son or daughter in the descriptions of the disorder, making the diagnosis more meaningful in terms of their daily experiences.

*P10: “It was completely consistent with issues like dramatic anxiety over abandonment and difficulty managing emotions.”*

*P14: “It’s also part of the disorder… for a few weeks, we might think our daughter is fine and then, suddenly, the symptoms reappear…”*.

Parents described how understanding the diagnosis profoundly influenced their identity as parents, their self-understanding, and their understanding of their child. They better comprehended the reasons behind their difficulties with their child, felt less anger, and consequently blamed their child less for challenging behaviors, which alleviated their own feelings of guilt.

*P15: “Understanding the pain of our loved one, realizing that if our loved one was unwell and showed significant anger, it was due to earlier experiences that led to this state, where the slightest thing could make him explode.”*

*P16: “We blamed ourselves a bit less for all her problems.”*

According to the parents, the diagnosis was only a step in the care process and needed to be followed by changes in treatment. It enabled parents to take action and seek specialized care for their loved one.

*P17: “I tried to find doctors who specialize in this disorder.”*

Parents also expressed a need for support from healthcare services.

*P8: “I felt the need for help… to find ways to maintain a connection despite the aggression.”*

### The pitfalls of receiving the diagnosis

#### Insufficient care, communication, and support for families following the diagnosis

For many parents, receiving the diagnosis did not lead to improved care.

*P1: “It didn’t allow her to get the appropriate care, because there wasn’t any available.”*

Parents often felt that healthcare services were not adequately informed about the disorder or did not provide care specific to BPD.

*P15: “In hospitals, clinics, or other settings, psychiatrists have not adapted their approaches to address the pathology.”*

Some parents expressed frustration over the continued lack of communication with mental healthcare services after receiving the diagnosis. They wished for greater recognition and appreciation of their role as caregivers by mental health professionals.

*P11: “Professionals should engage with the patient’s support system to aid their progress.”*

Parents described a paradoxical view regarding the role assigned to them in their child’s care by professionals. On one hand, they were expected to be actively involved in caring for their sons and daughters; on the other, they felt dismissed and insufficiently informed by psychiatrists or supported by healthcare services.

#### The risk of stigmatization

For many parents, borderline personality disorder (BPD) is not well recognized by the general public, leading to a trivialization of their challenges because of widespread ignorance about the disorder.

*P1: “The difficulty is, if I tell people it is a borderline disorder, it doesn’t have a big effect on them, if I tell them schizophrenic, they will say ah yes, that’s serious… but borderline everyone says yes, well…”*.

*P2: “All these illnesses have been stigmatized. Mental illness has become… the terminology schizo bipo… ah well you’re bipo, you’re schizo, you’re borderline, you’re completely crazy…”*.

Parents also encountered stigmatizing comments that contributed to their reluctance to discuss the diagnosis, even with family or friends, fearing judgment.

*P13: “These psychiatric illnesses are always perceived strangely in families.”*

Moreover, parents perceived a stigma within the healthcare environment, which manifested as difficulties in accessing care for their child and a generally pessimistic discourse about the disorder.

Participation in the Family Connections program, however, provided significant relief. It helped reduce their social isolation by connecting them with other parents who were experiencing similar challenges. These meetings were valuable as they shared difficulties and strategies.

*P3: “I found a group where we finally knew what we were talking about, and it felt really good.”*

*P19: “Listening to parents talk about taking action, hearing them say that things are getting better.”*

## Discussion

A significant finding of this research is that parents of individuals with BPD often face considerable delays before receiving a diagnosis, regardless of whether the individual is a minor or an adult. Additionally, the communication of the diagnosis typically occurs outside of dedicated formal consultations, lacking complete and adequate information, and without opportunities for parents to ask questions.

Parents emphasized the importance of obtaining a diagnosis to better understand and accept their child’s difficulties, thereby improving their child’s care. Additionally, parents expressed a desire for more support from healthcare services. Our findings suggest that the Family Connections program serves to address critical gaps in mental health services, providing support in areas such as the assimilation and acceptance of the diagnosis, recognition of their role as caregivers, and the provision of clear information and moral support.

Our results concerning the delay in receiving a diagnosis and the conditions of its communication align with existing literature on BPD [11, 28, 43, 44] as well as studies on the communication of other psychiatric disorders to families, such as schizophrenia [45] and bipolar disorder [46]. These studies emphasize that families wish to be involved early in the treatment process and be informed about the diagnostic proceedings. They appear to prefer tentative diagnostic hypotheses—even if these may later be disproven—over a prolonged absence of diagnosis, which diminishes their confidence in the care provided. A systematic review examining how patients, clinicians, and families experience psychiatric diagnoses revealed divergent perspectives on the timing of diagnosis [47]: patients and families felt the diagnosis was delayed excessively, whereas clinicians indicated a need for more time due to various factors, including the challenge of differentiating disorders with overlapping symptoms, determining when symptoms reach clinical significance, and managing complications from fluctuating symptoms. Furthermore, diagnosing BPD in young patients is controversial, as adolescence involves significant developmental changes in personality. It is challenging to define a stable personality disorder within this constantly evolving process. Some clinicians believe that diagnosing during this phase could perpetuate the disorder, stigmatize adolescents, and risk the medicalization of their development [48]. This underscores the need for open, collaborative dialogue among patients, families, and professionals regarding preferences and expectations for the diagnostic process. It is crucial to convey that diagnostic uncertainty can be transparently shared in collaborative care and does not impede treatment. We should also emphasize that early care can prevent the disorder from lasting over time.

Our findings on the necessity for high-quality communication about the diagnosis among all stakeholders resonate with what Fallowfield and Jenkins have shown more generally regarding the communication of bad news in medicine [49]: “If bad news is poorly communicated, it can cause confusion and lasting distress. Conversely, well-delivered announcements can aid in understanding and accepting the condition, facilitating adjustment.” Our research indicates that a lack of clear information has left parents confused and misunderstood about the diagnosis, leading to feelings of helplessness. Furthermore, in the absence of adequate information, caregivers often resort to conducting their research online, typically without guidance, which may expose them to inaccurate and pessimistic information about the disorder, exacerbating their distress [29, 43].

Many advantages of communicating a diagnosis to parents are highlighted in both our results and the literature. Communicating a diagnosis allows “the disorder to exist” in the eyes of parents and society, thereby legitimizing and validating the suffering of both the person with BPD and their parents. As Jutel and Nettleton have stated [50], “Being diagnosed gives permission to be ill (…) The complaint is recognized as an illness, and the individual will be treated instead of blamed.” The parents in our study described a positive social impact associated with the recognition of the illness, as it potentially allows access to compensation, tools to alleviate the consequences of the illness, and explanations for why the individual differs from the norm [51]. Moreover, the diagnosis enables parents who were passively affected by the disorder to take action [6]. This action was reflected in our study by the numerous searches for explanations about the disorder, care for their child, and support for themselves following the diagnosis. Other studies have shown how communication of the diagnosis is perceived as a pathway to access care [50, 52, 53]. Finally, the diagnosis leads to “biographical disruption,” which is interpreted as a process involving significant changes in an individual’s life and biography—altering the perception and interpretation of the life course [54].

Parents also addressed the pitfalls of receiving a diagnosis. Our results underlined the parents’ fear that the diagnosis might “reduce” and “confine” their child’s identity. According to Piot [6], “the way the patient is viewed changes from the moment the diagnosis is made.” Psychiatric diagnoses are often stigmatizing and risk increasing the patient’s psychological suffering, leading to social rejection and devaluation [53]. The parents in our study mentioned their social isolation in connection with the stigma associated with the disorder and their reluctance to discuss it outside the family circle. They emphasized the general population’s lack of knowledge about BPD [44]. As noted by parents, the diagnosis of BPD is particularly stigmatized within healthcare settings. Patients with BPD may be perceived as “manipulative and difficult” [55]. Recent studies have shown that nurses exhibit less empathy toward patients with BPD than those with other mental disorders [56]. Paradoxically, patients with BPD are described as both “intractable” and “not really sick” [23].

Finally, the experience of receiving the diagnosis became an integral part of the overall care experience. Parents interviewed in this study expressed dissatisfaction in several areas: access to care, communication with services, parental involvement in care, and support for parents. This dissatisfaction persisted even after the diagnosis was communicated, reflecting similar findings in the literature [27, 43, 44, 57, 58]. Additionally, caregivers may feel stigmatized by professionals who, in their perception, hold them responsible for the development of symptoms or view them as overly intrusive in their caregiving [11]. Professionals have also examined family involvement from their perspective reporting a lack of understanding of the family’s needs, inadequate skills to address those needs due to insufficient training, time constraints, and a lack of comprehensive care within healthcare services [59]. Recent findings suggest that training healthcare practitioners in BPD educational interventions can improve attitudes and practices toward individuals with BPD [60, 61]. We believe that enhancing professionals’ knowledge about BPD can also positively influence their approach to the relatives of individuals with BPD and facilitate improved communication within families. Additionally, involving families in care may have a therapeutic effect on the patient’s relatives, assisting them in navigating autonomy and individual agency. However, achieving this balance poses a challenge, as meeting the desires of parents to be involved must be balanced against the need to support the patient’s gradual development of autonomy and independence [62].

### Diagnostic disclosure

Our results reinforce aspects already documented in the literature [13, 15, 17, 63, 64] and bring to light specific issues concerning diagnostic disclosure that need to be addressed in clinical practice.

Parents expressed a desire to be involved in the diagnostic process earlier, even if the diagnosis remains tentative. This desire may stem from feelings of profound loneliness and abandonment. The absence of communication is particularly challenging and diminishes their trust in the healthcare system. They perceive a lack of diagnosis or a misdiagnosis as indicative of substandard care, which further erodes their trust.

These findings suggest that clinicians should involve both the patient and their family early in the diagnostic process, and consider the timing of disclosure to meet the expectations and needs of both parties. Practically, this involvement could take the form of a collaborative and progressive process with both patients and their families.

Furthermore, although guidelines recommend disclosure during a scheduled consultation—to ensure clinicians have sufficient time to explain the disorder clearly, address the emotional responses of the patient and their family, and answer their questions—most parents reported that disclosures were made in informal settings with limited information provided.

It is also advisable to describe symptoms by linking them to the personal experiences of patients and their families, rather than presenting a list of generic symptoms. This approach helps patients and their relatives to better recognize and understand their own experiences [47].

Parents also emphasized the importance of receiving information about therapeutic options and prognosis simultaneously, enabling them to envision a future and maintain hope. This suggests that healthcare professionals should discuss therapeutic options, expected treatment outcomes, and provide a comprehensive prognosis, outlining all potential scenarios from best to worst.

Finally, addressing the general perceptions of psychiatric disorders, particularly BPD, is essential. Participants in our study, having no prior knowledge of BPD, did not hold specific preconceived notions about it. However, without adequate support and regular follow-ups after disclosure, prejudices and self-stigmatization can emerge. Healthcare services could, for instance, integrate psychoeducational interventions like the Family Connections program, which has been developed specifically for family members [65].

### Limitations

This qualitative study had several limitations. First, it was conducted in France, and caution should be exercised when applying our findings to other countries, as they are specific to the French sociocultural context, and particularly to the French mental healthcare system. Second, the parents were recruited through the Family Connections program, meaning they possessed more knowledge and insights about the mental disorder affecting their child than parents not in the program. This may limit the transferability of our results to contexts where parents have not received similar training. A similar study should be conducted in diverse care settings, such as psychiatric outpatient treatment centers, to validate these findings further. Third, our research team consisted exclusively of psychiatrists, who bring a medical perspective to mental health issues. Including researchers from non-medical backgrounds or those with personal or familial experience with BPD could provide additional perspectives on the diagnosis of the disorder.

## Conclusion

This study highlights that communicating a BPD diagnosis to parents is a critical step in the treatment process. Parents typically desire early involvement in the diagnostic process and seek comprehensive and clear information when the diagnosis is made.

The more parents understand the challenges faced by their child, the better they can contribute to their care. However, the experience of receiving a diagnosis often correlates with a general dissatisfaction regarding the interaction between parents and mental health services, which are perceived as inadequate. This study underscores the necessity for psychiatrists to improve communication of the diagnosis and to engage parents more extensively in their child’s care. The Family Connections program is identified as a valuable support resource that should be readily available to parents of individuals diagnosed with BPD.

### Electronic supplementary material

Below is the link to the electronic supplementary material.

Supplementary Material 1

Supplementary Material 2

Supplementary Material 3

## Data Availability

Data will be provided upon request to the corresponding author for reasonable use.

## Abbreviations

BPD: Borderline personality disorder
