# Supplementary material for: The lived experience of French parents concerning the diagnosis of their children with borderline personality disorder
Source: Borderline Personal Disord Emot Dysregul. 2024 Jul 1;11:13. doi: 10.1186/s40479-024-00258-z (PMC11215819; doi:10.1186/s40479-024-00258-z)
Supplement: Supplementary file 1 — Supplementary Material 1 [file 40479_2024_258_MOESM1_ESM.docx]

**Table S1**. Characteristics of each participant

| Parents | Sex | Age | Family connection program | Son or daughter with BPD | | | | | | | | | |
| --- | --- | --- | --- | --- | --- | --- | --- | --- | --- | --- | --- | --- | --- |
|  |  |  |  | sex | age | Age at diagnosis | Living with parents | Social status | Follow-up by mental health professionals | pharmacotherapy | Inpatient treatment | Protective measures | National disability allowance |
| P1 | F | 54 | 2017 | F | 30 | 22 | No | Unemployed | None | No | > 10 | None | Yes |
| P2 | F | 63 | 2017 | F | 32 | 24 | No | Unemployed | None | Yes | 5 | None | No |
| P3 | F | 64 | 2020 | M | 25 | 24 | No | Student | None | No | 0 | None | No |
| P4 | M | 61 | 2018 | F | 24 | 19 | No | Unemployed | Psychiatrist | Yes | 1-5 | None | No |
| P5 & P6 | M+F | 61 & 62 | 2020 | F | 26 | 24 | No | Unemployed | None | Yes | 5 | None | No |
| P7 | F | 50 | 2020 | F | 27 | 25 | No | Unqualified employment | Psychiatrist Psychologist | Yes | 2 | None |  |
| P8 | F | 61 | 2020 | F | 34 | 24 | No | Unqualified employment | Psychiatrist  Psychologist | Yes | 6-9 | Reinforced curatorship | Yes |
| P9 | F | 50 | 2020 | F | 23 | 19 | No | Unqualified employment | None | No | 6-9 | None | No |
| P10 | F | 64 | 2019 | F | 28 | 24 | Yes | Unemployed | None | No | 0 | None | Yes |
| P11 | F | 63 | 2017 | F | 27 | 23 | No | Unemployed | Psychiatrist | Yes | 1 | Simple curatorship | Yes |
| P12 | M | 65 | 2019 | F | 34 | 24 | No | Unqualified employment | Psychiatrist  Psychologist | Yes | 10 | Reinforced curatorship | Yes |
| P13 | F | 47 | 2019 | F | 19 | 15 | Yes | Student | Psychiatrist | Yes | > 10 | None | Yes |
| P14 | M | 51 | 2020 | F | 25 | 24 | No | Unqualified employment | Psychiatrist | Yes | 2 | None | No |
| P15 | F | 52 | 2020 | F | 20 | 18 | Yes | Unemployed | Psychiatrist  Psychologist | Yes | 6-9 | None | Yes |
| P16 | F | 46 | 2020 | F | 15 | 14 | Yes | School refusal | Psychiatrist | Yes | 2 | None | No |
| P17 | F | 48 | 2021 | F | 20 | 17 | Yes | Student | Psychiatrist  Psychologist | Yes | 4 | None | No |
| P18 | F | 61 | 2019 | F | 33 | 23 | No | Unemployed | Psychologist | Yes | 5 | Reinforced curatorship | Yes |
| P19 | M | 69 | 2019 | F | 33 | 20 | No | Unemployed | Psychiatrist  Psychologist | Yes | 4 | Reinforced curatorship | Yes |
| P20 | F | 57 | 2020 | F | 28 | 24 | Yes | Student | Psychiatrist | Yes | 8 | None | No |
| P21 | M | 57 | 2022 | F | 25 | 21 | Yes | Unemployed | Psychiatrist | Yes | 5 | None | No |
