# Supplementary material for: The lived experience of French parents concerning the diagnosis of their children with borderline personality disorder
Source: Borderline Personal Disord Emot Dysregul. 2024 Jul 1;11:13. doi: 10.1186/s40479-024-00258-z (PMC11215819; doi:10.1186/s40479-024-00258-z)
Supplement: Supplementary file 2 — Supplementary Material 2 [file 40479_2024_258_MOESM2_ESM.docx]

**Table S2.** Original French quotations

| **The long and difficult road to diagnosis** |
| --- |
| *The delay in getting a diagnosis* |
| P2: “They spoke vaguely about a borderline disorder, then they went back to bipolarity.”  **« Ils ont parlé très vaguement d’état limite et après ils repartent sur bipolarité. »** |
| P4: “Over four years, I can't remember a single time a diagnosis was even considered as a hypothesis…”  **« En quatre ans, je ne me souviens pas d’une seule fois ou un diagnostic aurait été envisagé même au titre d’hypothèse … »** |
| P1: “The psychiatrist said she was giving medication to test things and she had nothing more to say.”  **« La psychiatrique nous disais qu’elle donnait des médicaments pour tester des choses et qu’elle n’avait pas rien à dire de plus. »** |
| P2: “We felt they had never read the DSMV…or the description of BPD…they were able to diagnose bipolarity, but not other disorders.”  **« On a l'impression qu'ils n'avaient jamais lu le DSM V... ou bien la description de la maladie TPL. Ils en étaient resté à...vaguement, ils arrivaient à diagnostiquer une bipolarité, mais pas au déjà quoi. »** |
| P1: “If she doesn’t have a diagnosis, why should she leave with an anti-depressant prescription.”  **« Si elle n’a pas de diagnostic, pourquoi elle est repartie avec une ordonnance d’antidépresseur. »** |
| *Informal diagnosis disclosure with few information* |
| P4: “He gave us some elements but without many details, at least I don't remember.”  **« Il nous a donné quelques éléments mais sans beaucoup de détails, ou alors je m’en rappelle plus. »** |
| P5: “It finally happened between two doors…the doctor told me: your daughter has a borderline personality disorder.”  **« C’est finalement entre deux portes…le docteur m’a dit : votre fille a un trouble de la personnalité limite. »** |
| P12: “I asked the psychiatrist what it meant because I had never heard of it.”  **« Et là, je lui ai demandé ce dont il s'agissait parce que je n'en avais jamais entendu parler. »** |
| P6: “I understand the doctor is very busy. He has a full waiting room all day. I understand that he does not have time to explain more.”  « **Je comprends que le docteur est très occupé, il a une salle pleine toute la journée, je comprends qu’il n’a pas le temps d’expliquer plus. »** |
| **Disclosing BPD diagnosis to parents: a necessary step** |
| *The immediate aftermath* |
| P12: “I was grateful to this professional for telling me… this person saved us in a way by giving words to a disorder, because finally someone told me what my daughter had.”  **« J'étais très reconnaissante envers ce professionnel de me l'avoir dit… Je pense que cette personne-là, elle nous a sauvé enfin d'une certaine manière, en mettant des mots sur un trouble parce qu'enfin on me disait ce que ma fille avait. »** |
| P4: “At the time, we didn't have many explanations, but we got leads to find documents and better understand the disorder.”  **« Sur le moment on n’avait pas beaucoup d’éléments d’explications mais on avait en tout cas des pistes pour se documenter et comprendre. »** |
| P8: “There are not many therapists, and there is no medication…borderline is not the easiest path.”  **« Il n’y a pas beaucoup de thérapeutes, et il n’y a pas de médicament…borderline n’est pas le chemin le plus facile. »** |
| P16: “I think I’m a little more careful in the way I approach her. That’s what Family Connections allowed me to do.”  **« Je crois que je suis un petit peu plus prudent dans ma façon de l’approcher, c’est ce que Connexions familiales m’a permis de faire. »** |
| P8: “This method allows us to better live and understand the illness of our loved ones and therefore to adjust our personal attitude... to the need for our loved ones to evolve.”  **« Cette méthode nous permet de mieux vivre et de comprendre la maladie de nos proches et donc**  **d'ajuster notre attitude personnelle… à la nécessité d'évolution de nos proches. »** |
| *A meaningful and helpful diagnosis?* |
| P5: “I hadn't fully integrated she was sick. It took me... a while.”  **« Moi, je n'avais pas intégré complètement qu'elle était malade. ça m'a pris... ça m'a pris du**  **Temps. »** |
| P10: “It was completely consistent with a difficulty in managing emotions, dramatic abandonment anxiety.”  **« Ca collait complètement avec une difficulté dans la gestion des émotions, une angoisse**  **d’abandon dramatique. »** |
| P14: “It’s also part of the disorder... for a few weeks, we can have the impression our daughter is fine and then, suddenly, the disorder comes back...”  **« Ca fait aussi partie de la maladie…on peut avoir l’impression pendant quelques semaines que**  **notre fille va très bien et puis d’un seul coup, d’un seul coup la maladie se ramène…»** |
| P15: “Understanding the pain of our loved one, understanding that if our loved one was not well and was showing significant excess anger, it was because there had been stages before that put him in this state of mind, and a little thing…it made him explode.”  **Déjà, apprendre la souffrance de notre proche, comprendre que Si notre proche n'était pas bien et faisait des excès de colère importants, c'était que il y avait eu des paliers auparavant qui le mettait dans cet état d'esprit et que le moindre truc et bah, ça le faisait exploser. »** |
| P16: “We blamed ourselves a little less for all her problems.”  **« On se culpabilise un petit peu moins pour tous les problèmes qui lui arrivent. »** |
| P17: “I tried to see if there were doctors… who specialized in this.”  **« J’ai essayé de voir s’il y a des docteurs…qui sont spécialisés là dedans. »** |
| P8: “I felt the need to be helped... to find keys to continue to maintain a link despite the violence.”  **« J’ai ressenti le besoin d’être aidé…pour trouver des outils pour continuer à maintenir un lien malgré la violence. »** |
| **The pitfalls of the diagnosis disclosure** |
| *Lack of care, communication, and support for families after the disclosure* |
| P1: "It didn't allow her to get the appropriate care, because there wasn't any.”  **« Ca ne l’a pas aidé à avoir des soins, car il n’y en avait pas. »** |
| P15*:* “In hospitals, clinics, or other places, psychiatrists have not adapted their way of doing things in relation to the pathology.”  **« Dans les hôpitaux, cliniques, ou autres, les psychiatres n’ont pas adaptés leur façon de faire les choses en lien avec la pathologie. »** |
| P11: “Professionals should rely on the patient's environment to help him/her progress.”  **« Les professionnels devraient prendre en compte l’environment du patient pour l’aider à progresser. »** |
| *The risk of stigma* |
| P1: “The difficulty is, if I tell people it is a borderline disorder, it doesn't have a big effect on them, if I tell them schizophrenic, they will say ah yes, that's serious... but borderline everyone says yes, well... “  **« la difficulté c’est que si aux gens je leur dis que c’est une maladie borderline ça ne leur fait pas**  **un grand effet, si je leur disais schizophrène là ils se diraient ah là oui ça c’est grave…mais borderline on va dire oui bon… »** |
| P2: “All these illnesses have been stigmatized. Mental illness has become... the terminology schizo bipo... ah well you're bipo, you're schizo, you're borderline, you're completely crazy.”  **« Toutes ces maladies ont été stigmatisées. La maladie mentale est devenue...la terminologie**  **schizo bipo...ah bah t’es bipo, t'es schizo, t'es borderline, t'es complètement frappadingue. »** |
| P13: “These psychiatric illnesses are always perceived strangely in families.”  **« Ces maladies psychiatriques sont toujours perçues bizarrement par les familles. »** |
| P3: “I found a group where we finally knew what we were talking about, and it felt really good.”  **« J’ai enfin trouvé un groupe où l’on sait de quoi on parle, et ça fait du bien. »** |
| P19: “Listening to parents talk about taking action, hearing them say that things are getting better.”  **« Entendre les parents parler des choses qu’ils ont pu mettre en place, les entendre dire que les choses allaient mieux. »** |
