# Supplementary material for: The lived experience of French parents concerning the diagnosis of their children with borderline personality disorder
Source: Borderline Personal Disord Emot Dysregul. 2024 Jul 1;11:13. doi: 10.1186/s40479-024-00258-z (PMC11215819; doi:10.1186/s40479-024-00258-z)
Supplement: Supplementary file 3 — Supplementary Material 3 [file 40479_2024_258_MOESM3_ESM.docx]

**Additional material on Family Connections**

**Background**

Family Connections is a program based on the principles of Dialectical Behavioral Therapy which is a type of cognitive-behavioral therapy developed by Marsha Linehan, that emphasizes the synthesis of acceptance and change-oriented strategies to address emotional dysregulation and interpersonal difficulties commonly experienced by individuals with BPD[1]. Incorporating Dialectical Behavioral Therapy principles into the program involves teaching skills such as mindfulness, distress tolerance, emotion regulation, and interpersonal effectiveness to help family members to better understand and cope with the challenges of living with and supporting someone with BPD [2]. Family Connections is organized into 6 modules, carried out over 12 weeks. This program is led by peers, in collaboration with healthcare professionals. The training modules for Family Connections are presented below:

1. Information about BPD (symptoms, disorder progression) and an overview of BPD research.
2. Psychoeducation: Development of the disorder, treatments, comorbidities, introduction to emotional dysregulation.
3. Skills to promote participant's emotional well-being: Emotion regulation individually and in relationships, mindfulness, letting go of judgments, reducing emotional vulnerability and reactivity.
4. Family relational skills: Letting go of reproaches and anger, radical acceptance.
5. Validation skills.
6. Problem-solving skills: Defining problems, collaborative problem-solving, dialectics of acceptance and change.

The Family Connections program has shown its effectiveness in several areas [3–9] : reducing caregiver burden with decreased psychological distress (decreased depression, decreased grief), increased family functioning, improved sense of mastery, and decreased symptoms for the relative with a BPD.

To our knowledge, there is only one qualitative study exploring caregivers' experiences related to their participation in Family Connections [7]. This study does not focus on a specific population of caregivers who have received a BPD diagnosis for their loved one but rather caregivers of adolescents with various diagnoses. This study highlights several benefits of Family Connections, including increased caregiver capacity to manage the difficulties of their loved ones, improved relationships with the relative, a better self-awareness, a sense of regaining control over the situation, and a sense of belonging to the group.

The second objective of our study was to evaluate the impact of the Family Connections program on the diagnosis experience using a qualitative methodology.

**Results**

The parents reported several positive effects regarding family connections training. After having gone through the program, parents had a better view and understanding of the BPD diagnosis and, as a direct consequence, noticed an improvement in communication and a calmer relationship with their son/daughter.

*P16: “I think I’m a little more careful in the way I approach her, that’s what Family Connections allowed me to do.”*

Parents also reported feeling less powerless after the program. They felt more capable of dealing with their son’s or daughter’s difficulties, especially in times of crisis, and had the impression of regaining control over the situation.

*P8: “This method allows us to better live and understand the illness of our loved ones and therefore to adjust our personal attitudes... to the need for our loved ones to evolve.”*

Parents also mentioned that Family Connections help them to “re-establishing boundaries”. In fact, they explained having difficulties saying "no" to their loved one because of the fear of suicidal behavior.

*P10 : "It's not you who will push her to suicide, it's her suffering, and it already exists »*

Among the skills acquired within Family Connections, validation and radical acceptance were considered essential for improving relationships with the loved one. Parents used these technics even after their participation in the group.

*P13 : « In fact, validating emotions is something that is extremely important in the relationships I have with her today."*

Participation in the Family Connections program also reduced their social isolation. They expressed a feeling of relief when meeting other parents living with a son/daughter with the same diagnosis and living with similar experiences, and felt they were finally meeting people who knew about the disorder. They were learning from the experiences of other parents who shared their difficulties and their initiatives.

*P3: “I found a group where we finally knew what we were talking about, and it felt really good.”*

*P19: “Listening to parents talk about taking action, hearing them say that things are getting better.”*

Some parents reported maintaining connections with other parents following their participation. Others expressed still being in contact with trainers who continued to provide support to them even after their participation.

*P11: "But we still have a monthly meeting with the Family Connections group. So, obviously, that's highly commendable, that's clear."*

**Discussion**

Our results highlight improvements in the experience of diagnosis following participation in the Family Connections training. Our study shows an enhancement in knowledge about the disorder, improvement in parents' abilities to manage difficulties related to the disorder, an increase in the sense of "mastery," and a more positive experience of the caregiving role. These elements are consistent with previous findings. According to the results of a qualitative study evaluating the impact of Family Connections, the training provides a new understanding of the relative's difficulties and an increase in the ability to cope with their difficulties [7]. The training also promotes acceptance of the illness and the caregiving role. Moreover, several studies have shown the effectiveness of the program in increasing the sense of "mastery" [3,5,6]. Improvement in communication is described by parents, and relationships between parents and the relative are more peaceful. These results are consistent with a qualitative study on the subject [7]. « Validating emotions » is a technique reported as essential by parents to facilitate communication with the relative [7]. An improvement in the relative's symptoms is also noted by parents. To our knowledge, only one quantitative study [9] has examined the impact of Family Connections on the improvement of the relative's symptoms. This study measured, using a validated scale (the Child Behavior Checklist), the relative's internalizing and externalizing symptoms. This study confirms a significant decrease in the relative's symptoms. Finally, meeting other parents helps to reduce social isolation, promotes understanding of the disorder, and the acquisition of skills through sharing parents' experiences, leading to a sense of belonging [7].

1. Stoffers-Winterling JM, Völlm BA, Rücker G, Timmer A, Huband N, Lieb K. Psychological therapies for people with borderline personality disorder. Cochrane Developmental, Psychosocial and Learning Problems Group, éditeur. Cochrane Database of Systematic Reviews [Internet]. 15 août 2012 [cité 21 août 2021]; Disponible sur: https://doi.wiley.com/10.1002/14651858.CD005652.pub2

2. Hoffman PD, Fruzzetti AE, Swenson CR. Dialectical behavior therapy--family skills training. Fam Process. 1999;38(4):399‑414.

3. Hoffman PD, Fruzzetti AE, Buteau E, Neiditch ER, Penney D, Bruce ML, et al. Family connections: a program for relatives of persons with borderline personality disorder. Fam Process. juin 2005;44(2):217‑25.

4. Flynn D, Kells M, Joyce M, Corcoran P, Herley S, Suarez C, et al. Family Connections versus optimised treatment-as-usual for family members of individuals with borderline personality disorder: non-randomised controlled study. Borderline Personal Disord Emot Dysregul. 2017;4:18.

5. Rajalin M, Wickholm-Pethrus L, Hursti T, Jokinen J. Dialectical behavior therapy-based skills training for family members of suicide attempters. Arch Suicide Res. 2009;13(3):257‑63.

6. Liljedahl SI, Kleindienst N, Wångby-Lundh M, Lundh LG, Daukantaitė D, Fruzzetti AE, et al. Family Connections in different settings and intensities for underserved and geographically isolated families: a non-randomised comparison study. Borderline Personal Disord Emot Dysregul. 2019;6:14.

7. Sheikhan NY, Wang K, Boritz T, Hawke LD, McMain S, Henderson J. Evaluating the effectiveness of the Family Connections program for caregivers of youth with mental health challenges, part II: A qualitative analysis. Health Expect. avr 2021;24(2):709‑18.

8. Hoffman PD, Fruzzetti AE, Buteau E. Understanding and engaging families: An education, skills and support program for relatives impacted by borderline personality disorder. Journal of Mental Health. 1 janv 2007;16(1):69‑82.

9. Boritz TZ, Sheikhan NY, Hawke LD, McMain SF, Henderson J. Evaluating the effectiveness of the Family Connections program for caregivers of youth with mental health challenges, part I: A quantitative analysis. Health Expect. avr 2021;24(2):578‑88.
